# 3-Amino-1-(4-meth­oxy­phen­yl)-9,10-dihydro­phenanthrene-2,4-dicarbonitrile

**DOI:** 10.1107/S1600536811033617

**Published:** 2011-08-27

**Authors:** Abdullah M. Asiri, Abdulrahman O. Al-Youbi, Hassan M. Faidallah, Seik Weng Ng, Edward R. T. Tiekink

**Affiliations:** a Chemistry Department, Faculty of Science, King Abdulaziz University, PO Box 80203, Jeddah, Saudi Arabia; b The Center of Excellence for Advanced Materials Research, King Abdulaziz University, PO Box 80203, Jeddah, Saudi Arabia; c Department of Chemistry, University of Malaya, 50603 Kuala Lumpur, Malaysia

## Abstract

In the title compound, C_23_H_17_N_3_O, significant deviations from planarity are evidenced. This is quanti­fied in the dihedral angles formed between the central amino-benzene ring and the benzene rings of the meth­oxy­benzene [67.93 (8)°] and 1,2-dihydro­naphthalene [28.27 (8)°] residues. In the crystal the amino-H atoms form hydrogen bonds to the meth­oxy-O atom and to one of the cyano-N atoms to generate a two-dimensional array with a zigzag topology that stacks along the (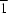 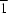 1) plane.

## Related literature

For background to the biological activity of related compounds, see: Aly *et al.* (1991 ▶); Al-Saadi *et al.* (2005 ▶); Rostom *et al.* (2011 ▶). For ring conformational analysis, see: Cremer & Pople (1975 ▶). For a related structure, see: Asiri *et al.* (2011 ▶).
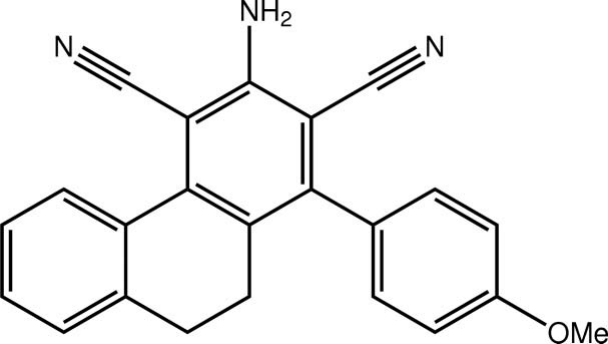


## Experimental

### 

#### Crystal data

C_23_H_17_N_3_O
*M* *_r_* = 351.40
Monoclinic, 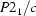

*a* = 9.0212 (4) Å
*b* = 22.1475 (8) Å
*c* = 9.3114 (4) Å
β = 110.410 (5)°
*V* = 1743.60 (12) Å^3^
*Z* = 4
Mo *K*α radiation
μ = 0.08 mm^−1^
*T* = 100 K
0.25 × 0.25 × 0.05 mm

#### Data collection

Agilent Technologies SuperNova Dual diffractometer with Atlas detector
Absorption correction: multi-scan (*CrysAlis PRO*; Agilent, 2010 ▶) *T*_min_ = 0.714, *T*_max_ = 1.000
8688 measured reflections
3890 independent reflections
2953 reflections with *I* > 2σ(*I*)
*R*_int_ = 0.030

#### Refinement

*R*[*F* ^2^ > 2σ(*F* ^2^)] = 0.048
*wR*(*F* ^2^) = 0.116
*S* = 1.04
3890 reflections
252 parameters
2 restraints
H atoms treated by a mixture of independent and constrained refinement
Δρ_max_ = 0.33 e Å^−3^
Δρ_min_ = −0.23 e Å^−3^

### 

Data collection: *CrysAlis PRO* (Agilent, 2010 ▶); cell refinement: *CrysAlis PRO*; data reduction: *CrysAlis PRO*; program(s) used to solve structure: *SHELXS97* (Sheldrick, 2008 ▶); program(s) used to refine structure: *SHELXL97* (Sheldrick, 2008 ▶); molecular graphics: *ORTEP-3* (Farrugia, 1997 ▶) and *DIAMOND* (Brandenburg, 2006 ▶); software used to prepare material for publication: *publCIF* (Westrip, 2010 ▶).

## Supplementary Material

Crystal structure: contains datablock(s) global, I. DOI: 10.1107/S1600536811033617/hg5084sup1.cif

Structure factors: contains datablock(s) I. DOI: 10.1107/S1600536811033617/hg5084Isup2.hkl

Supplementary material file. DOI: 10.1107/S1600536811033617/hg5084Isup3.cml

Additional supplementary materials:  crystallographic information; 3D view; checkCIF report

## Figures and Tables

**Table 1:** Hydrogen-bond geometry (Å, °)

*D*—H⋯*A*	*D*—H	H⋯*A*	*D*⋯*A*	*D*—H⋯*A*
N2—H1⋯O1^i^	0.89 (1)	2.21 (1)	3.0307 (19)	154 (2)
N2—H2⋯N1^ii^	0.88 (1)	2.33 (1)	3.115 (2)	149 (2)

Symmetry codes: (i) 
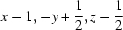
; (ii) 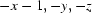.

**Fig. 1.**
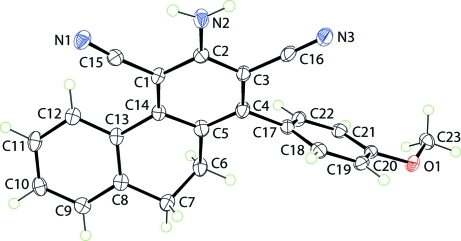
The molecular structure of (I) showing the atom-labelling scheme and displacement ellipsoids at the 50% probability level.

**Fig. 2.**
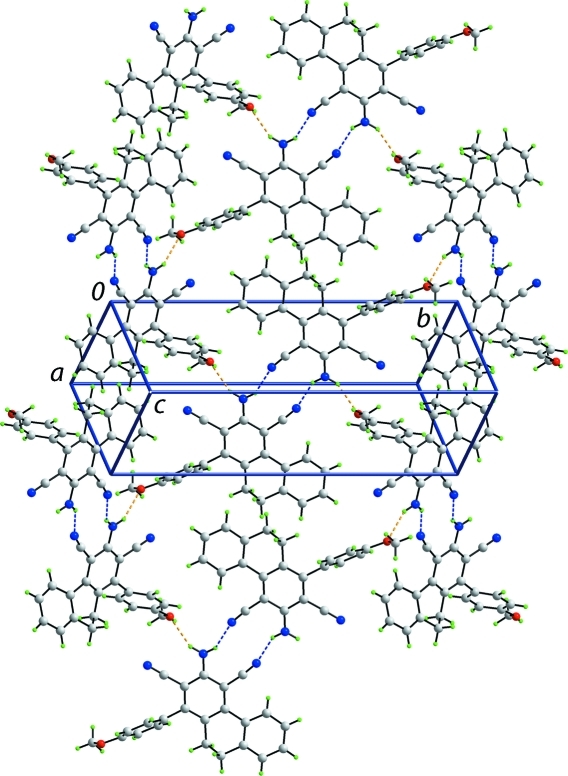
Supramolecular array in (I) viewed towards the (11 1) plane. The N—H···O and N—H···.N hydrogen bonds are shown as orange and blue dashed lines, respectively.

## supplementary crystallographic information

### Comment

The study of the title compound (I) was motivated by recent reports of the biological activity of related compounds (Aly *et al.*, 1991; Al-Saadi *et al.*, 2005; Rostom *et al.*, 2011) and allied crystal structure investigations (Asiri *et al.*, 2011).

The structure of (I), Fig. 1, is isostructural with the derivative in which the methoxybenzene group in (I) is substituted for a 2*H*-1,3-benzodioxol-5-yl group (Asiri *et al.*, 2011). With respect to the amino-benzene ring, the benzene rings of the methoxybenzene and 1,2-dihydronaphthalene residues form dihedral angles of 67.93 (8) and 28.27 (8) °, respectively, indicating non-planarity in the molecule. In the 1,2-dihydronaphthalene residue, the cyclohexa-1,3-diene ring has a distorted half-chair conformation as defined by the following parameters (Cremer & Pople, 1975): q_2_ = 0.5166 (18) Å, φ_2_ = 84.4 (2) °, q_3_ = 0.1891 (19) Å, and puckering amplitude Q = 0.5501 (19) Å.

In the crystal structure, supramolecular arrays with zigzag topology and running parallel to the (11 1) plane are formed through *N*—*H*···*O*(methoxy) and *N*—*H*···*N*(cyano) hydrogen bonding, Table 1 and Fig. 2.

### Experimental

A mixture of the 4-anisaldehyde (1.36 g,10 mmol), 1-tetralone (1.46 g, 10 mmol), ethyl cyanoacetate (1.1 g, 10 mmol) and ammonium acetate (6.2 g, 80 mmol) in absolute ethanol (50 ml) was refluxed for 6 h. The reaction mixture was allowed to cool and the precipitate that formed was filtered, washed with water, dried and recrystallized from DMF; *M*.pt.: 487–488 K.

### Refinement

Carbon-bound H-atoms were placed in calculated positions [C—H 0.95 to 0.99 Å, *U*_iso_(H) 1.2 to 1.5*U*_eq_(C)] and were included in the refinement in the riding model approximation. The amino-H atoms were located in a difference Fourier map, and subsequently refined with N—H = 0.88±0.01 Å.

### Crystal data

**Table:**

C_23_H_17_N_3_O	*F*(000) = 736
*M**_r_* = 351.40	*D*_x_ = 1.339 Mg m^−^^3^
Monoclinic, *P*2_1_/*c*	Mo *K*α radiation, λ = 0.71073 Å
Hall symbol: -P 2ybc	Cell parameters from 3338 reflections
*a* = 9.0212 (4) Å	θ = 2.3–29.3°
*b* = 22.1475 (8) Å	µ = 0.08 mm^−^^1^
*c* = 9.3114 (4) Å	*T* = 100 K
β = 110.410 (5)°	Plate, orange
*V* = 1743.60 (12) Å^3^	0.25 × 0.25 × 0.05 mm
*Z* = 4	

### Data collection

**Table:**

Agilent Technologies SuperNova Dual diffractometer with Atlas detector	3890 independent reflections
Radiation source: SuperNova (Mo) X-ray Source	2953 reflections with *I* > 2σ(*I*)
mirror	*R*_int_ = 0.030
Detector resolution: 10.4041 pixels mm^-1^	θ_max_ = 27.5°, θ_min_ = 2.4°
ω scan	*h* = −9→11
Absorption correction: multi-scan (*CrysAlis PRO*; Agilent, 2010)	*k* = −26→28
*T*_min_ = 0.714, *T*_max_ = 1.000	*l* = −12→11
8688 measured reflections	

### Refinement

**Table:**

Refinement on *F*^2^	Primary atom site location: structure-invariant direct methods
Least-squares matrix: full	Secondary atom site location: difference Fourier map
*R*[*F*^2^ > 2σ(*F*^2^)] = 0.048	Hydrogen site location: inferred from neighbouring sites
*wR*(*F*^2^) = 0.116	H atoms treated by a mixture of independent and constrained refinement
*S* = 1.04	*w* = 1/[σ^2^(*F*_o_^2^) + (0.0431*P*)^2^ + 0.7237*P*] where *P* = (*F*_o_^2^ + 2*F*_c_^2^)/3
3890 reflections	(Δ/σ)_max_ < 0.001
252 parameters	Δρ_max_ = 0.33 e Å^−^^3^
2 restraints	Δρ_min_ = −0.23 e Å^−^^3^

### Special details

**Table:**

Geometry. All e.s.d.'s (except the e.s.d. in the dihedral angle between two l.s. planes) are estimated using the full covariance matrix. The cell e.s.d.'s are taken into account individually in the estimation of e.s.d.'s in distances, angles and torsion angles; correlations between e.s.d.'s in cell parameters are only used when they are defined by crystal symmetry. An approximate (isotropic) treatment of cell e.s.d.'s is used for estimating e.s.d.'s involving l.s. planes.
Refinement. Refinement of *F*^2^ against ALL reflections. The weighted *R*-factor *wR* and goodness of fit *S* are based on *F*^2^, conventional *R*-factors *R* are based on *F*, with *F* set to zero for negative *F*^2^. The threshold expression of *F*^2^ > σ(*F*^2^) is used only for calculating *R*-factors(gt) *etc*. and is not relevant to the choice of reflections for refinement. *R*-factors based on *F*^2^ are statistically about twice as large as those based on *F*, and *R*- factors based on ALL data will be even larger.

### Fractional atomic coordinates and isotropic or equivalent isotropic displacement parameters (Å^2^)

**Table:**

	*x*	*y*	*z*	*U*_iso_*/*U*_eq_	
O1	0.50780 (13)	0.32058 (5)	0.20429 (14)	0.0233 (3)	
N1	−0.35267 (17)	−0.04178 (6)	0.09331 (17)	0.0253 (3)	
N2	−0.31052 (17)	0.09057 (7)	−0.04975 (18)	0.0273 (4)	
H1	−0.341 (2)	0.1248 (6)	−0.101 (2)	0.029 (5)*	
H2	−0.383 (2)	0.0640 (8)	−0.048 (2)	0.040 (6)*	
N3	−0.12484 (19)	0.22536 (7)	−0.09041 (19)	0.0348 (4)	
C1	−0.11308 (18)	0.02880 (7)	0.13513 (19)	0.0181 (3)	
C2	−0.15970 (18)	0.08249 (7)	0.04721 (19)	0.0191 (4)	
C3	−0.04407 (19)	0.12715 (7)	0.06686 (19)	0.0194 (4)	
C4	0.11146 (19)	0.11967 (8)	0.1705 (2)	0.0221 (4)	
C5	0.15320 (19)	0.06722 (8)	0.2563 (2)	0.0222 (4)	
C6	0.31513 (19)	0.05910 (8)	0.3770 (2)	0.0245 (4)	
H6A	0.3719	0.0982	0.3975	0.029*	
H6B	0.3777	0.0302	0.3403	0.029*	
C7	0.2964 (2)	0.03549 (7)	0.5226 (2)	0.0221 (4)	
H7A	0.4017	0.0295	0.6024	0.027*	
H7B	0.2372	0.0651	0.5613	0.027*	
C8	0.20844 (19)	−0.02353 (7)	0.4888 (2)	0.0201 (4)	
C9	0.2469 (2)	−0.07051 (8)	0.5946 (2)	0.0219 (4)	
H9	0.3289	−0.0650	0.6908	0.026*	
C10	0.1678 (2)	−0.12526 (8)	0.5624 (2)	0.0235 (4)	
H10	0.1918	−0.1563	0.6375	0.028*	
C11	0.0535 (2)	−0.13428 (8)	0.4195 (2)	0.0242 (4)	
H11	0.0014	−0.1722	0.3953	0.029*	
C12	0.01471 (19)	−0.08839 (7)	0.3118 (2)	0.0213 (4)	
H12	−0.0623	−0.0955	0.2134	0.026*	
C13	0.08722 (18)	−0.03175 (7)	0.34554 (19)	0.0182 (3)	
C14	0.04050 (18)	0.02098 (7)	0.24062 (19)	0.0183 (3)	
C15	−0.24040 (19)	−0.01280 (7)	0.11644 (19)	0.0206 (4)	
C16	−0.0873 (2)	0.18193 (8)	−0.0198 (2)	0.0235 (4)	
C17	0.22517 (18)	0.17059 (7)	0.18816 (19)	0.0198 (4)	
C18	0.20113 (19)	0.22527 (8)	0.25000 (19)	0.0219 (4)	
H18	0.1168	0.2292	0.2884	0.026*	
C19	0.29915 (19)	0.27411 (8)	0.25614 (19)	0.0200 (4)	
H19	0.2823	0.3112	0.2993	0.024*	
C20	0.42161 (18)	0.26902 (7)	0.19956 (18)	0.0186 (3)	
C21	0.45114 (19)	0.21406 (8)	0.14320 (19)	0.0215 (4)	
H21	0.5378	0.2098	0.1084	0.026*	
C22	0.35293 (19)	0.16540 (8)	0.13821 (19)	0.0217 (4)	
H22	0.3733	0.1277	0.0999	0.026*	
C23	0.5989 (2)	0.32247 (8)	0.1054 (2)	0.0254 (4)	
H23A	0.6552	0.3611	0.1186	0.038*	
H23B	0.5283	0.3184	−0.0013	0.038*	
H23C	0.6755	0.2893	0.1314	0.038*	

### Atomic displacement parameters (Å^2^)

**Table:**

	*U*^11^	*U*^22^	*U*^33^	*U*^12^	*U*^13^	*U*^23^
O1	0.0230 (6)	0.0200 (6)	0.0264 (7)	−0.0091 (5)	0.0079 (5)	−0.0019 (5)
N1	0.0189 (7)	0.0213 (8)	0.0318 (8)	−0.0017 (6)	0.0039 (6)	0.0000 (6)
N2	0.0174 (7)	0.0234 (8)	0.0343 (9)	−0.0044 (6)	0.0004 (7)	0.0086 (7)
N3	0.0357 (9)	0.0216 (8)	0.0331 (9)	−0.0061 (7)	−0.0056 (7)	0.0032 (7)
C1	0.0162 (8)	0.0153 (8)	0.0234 (8)	−0.0010 (6)	0.0078 (7)	−0.0013 (6)
C2	0.0171 (8)	0.0200 (8)	0.0202 (8)	−0.0002 (7)	0.0063 (7)	−0.0007 (7)
C3	0.0189 (8)	0.0161 (8)	0.0216 (8)	−0.0005 (6)	0.0052 (7)	0.0010 (6)
C4	0.0192 (8)	0.0194 (9)	0.0263 (9)	−0.0033 (7)	0.0061 (7)	0.0005 (7)
C5	0.0162 (8)	0.0229 (9)	0.0257 (9)	−0.0003 (7)	0.0050 (7)	0.0033 (7)
C6	0.0146 (8)	0.0218 (9)	0.0339 (10)	−0.0008 (7)	0.0043 (7)	0.0060 (8)
C7	0.0179 (8)	0.0183 (8)	0.0268 (9)	0.0009 (7)	0.0036 (7)	0.0005 (7)
C8	0.0170 (8)	0.0184 (8)	0.0268 (9)	0.0033 (7)	0.0101 (7)	0.0003 (7)
C9	0.0205 (8)	0.0222 (9)	0.0235 (9)	0.0052 (7)	0.0083 (7)	0.0009 (7)
C10	0.0245 (9)	0.0181 (9)	0.0306 (10)	0.0049 (7)	0.0130 (8)	0.0060 (7)
C11	0.0198 (8)	0.0162 (8)	0.0377 (10)	0.0015 (7)	0.0114 (8)	0.0026 (7)
C12	0.0156 (8)	0.0200 (9)	0.0278 (9)	0.0017 (7)	0.0071 (7)	0.0006 (7)
C13	0.0140 (7)	0.0153 (8)	0.0266 (9)	0.0034 (6)	0.0090 (7)	0.0023 (7)
C14	0.0173 (8)	0.0164 (8)	0.0224 (8)	0.0014 (6)	0.0083 (7)	0.0004 (7)
C15	0.0194 (8)	0.0186 (8)	0.0220 (9)	0.0039 (7)	0.0049 (7)	0.0016 (7)
C16	0.0193 (8)	0.0200 (9)	0.0251 (9)	−0.0059 (7)	0.0001 (7)	−0.0024 (7)
C17	0.0160 (8)	0.0184 (8)	0.0202 (8)	−0.0019 (7)	0.0002 (7)	0.0034 (7)
C18	0.0174 (8)	0.0254 (9)	0.0214 (9)	−0.0015 (7)	0.0050 (7)	0.0027 (7)
C19	0.0193 (8)	0.0191 (8)	0.0191 (8)	−0.0005 (7)	0.0037 (7)	−0.0025 (7)
C20	0.0166 (8)	0.0189 (8)	0.0165 (8)	−0.0049 (7)	0.0013 (6)	0.0013 (6)
C21	0.0194 (8)	0.0248 (9)	0.0200 (8)	−0.0015 (7)	0.0065 (7)	0.0002 (7)
C22	0.0229 (8)	0.0171 (8)	0.0228 (9)	0.0002 (7)	0.0049 (7)	−0.0011 (7)
C23	0.0219 (9)	0.0271 (10)	0.0261 (9)	−0.0061 (7)	0.0072 (7)	0.0052 (7)

### Geometric parameters (Å, °)

**Table:**

O1—C20	1.3735 (19)	C8—C13	1.411 (2)
O1—C23	1.433 (2)	C9—C10	1.386 (2)
N1—C15	1.154 (2)	C9—H9	0.9500
N2—C2	1.357 (2)	C10—C11	1.384 (2)
N2—H1	0.889 (9)	C10—H10	0.9500
N2—H2	0.883 (9)	C11—C12	1.385 (2)
N3—C16	1.147 (2)	C11—H11	0.9500
C1—C14	1.402 (2)	C12—C13	1.399 (2)
C1—C2	1.421 (2)	C12—H12	0.9500
C1—C15	1.435 (2)	C13—C14	1.486 (2)
C2—C3	1.402 (2)	C17—C22	1.390 (2)
C3—C4	1.408 (2)	C17—C18	1.390 (2)
C3—C16	1.434 (2)	C18—C19	1.385 (2)
C4—C5	1.385 (2)	C18—H18	0.9500
C4—C17	1.494 (2)	C19—C20	1.384 (2)
C5—C14	1.414 (2)	C19—H19	0.9500
C5—C6	1.512 (2)	C20—C21	1.388 (2)
C6—C7	1.517 (2)	C21—C22	1.386 (2)
C6—H6A	0.9900	C21—H21	0.9500
C6—H6B	0.9900	C22—H22	0.9500
C7—C8	1.504 (2)	C23—H23A	0.9800
C7—H7A	0.9900	C23—H23B	0.9800
C7—H7B	0.9900	C23—H23C	0.9800
C8—C9	1.391 (2)		
			
C20—O1—C23	116.67 (13)	C9—C10—H10	120.3
C2—N2—H1	121.6 (13)	C10—C11—C12	120.38 (16)
C2—N2—H2	118.4 (14)	C10—C11—H11	119.8
H1—N2—H2	119.1 (19)	C12—C11—H11	119.8
C14—C1—C2	121.91 (14)	C11—C12—C13	121.00 (16)
C14—C1—C15	124.01 (15)	C11—C12—H12	119.5
C2—C1—C15	113.81 (14)	C13—C12—H12	119.5
N2—C2—C3	121.41 (15)	C12—C13—C8	118.40 (15)
N2—C2—C1	121.35 (15)	C12—C13—C14	123.62 (15)
C3—C2—C1	117.23 (14)	C8—C13—C14	117.96 (14)
C2—C3—C4	121.63 (15)	C1—C14—C5	118.82 (15)
C2—C3—C16	118.66 (15)	C1—C14—C13	122.83 (14)
C4—C3—C16	119.70 (15)	C5—C14—C13	118.19 (14)
C5—C4—C3	119.95 (15)	N1—C15—C1	173.19 (17)
C5—C4—C17	122.08 (15)	N3—C16—C3	178.65 (19)
C3—C4—C17	117.93 (15)	C22—C17—C18	118.55 (15)
C4—C5—C14	120.44 (15)	C22—C17—C4	121.21 (15)
C4—C5—C6	121.76 (15)	C18—C17—C4	120.19 (15)
C14—C5—C6	117.65 (15)	C19—C18—C17	120.51 (15)
C5—C6—C7	109.01 (14)	C19—C18—H18	119.7
C5—C6—H6A	109.9	C17—C18—H18	119.7
C7—C6—H6A	109.9	C20—C19—C18	120.22 (15)
C5—C6—H6B	109.9	C20—C19—H19	119.9
C7—C6—H6B	109.9	C18—C19—H19	119.9
H6A—C6—H6B	108.3	O1—C20—C19	115.98 (14)
C8—C7—C6	109.13 (14)	O1—C20—C21	124.07 (15)
C8—C7—H7A	109.9	C19—C20—C21	119.94 (15)
C6—C7—H7A	109.9	C22—C21—C20	119.35 (15)
C8—C7—H7B	109.9	C22—C21—H21	120.3
C6—C7—H7B	109.9	C20—C21—H21	120.3
H7A—C7—H7B	108.3	C21—C22—C17	121.31 (15)
C9—C8—C13	119.53 (15)	C21—C22—H22	119.3
C9—C8—C7	121.24 (15)	C17—C22—H22	119.3
C13—C8—C7	119.21 (15)	O1—C23—H23A	109.5
C10—C9—C8	121.23 (16)	O1—C23—H23B	109.5
C10—C9—H9	119.4	H23A—C23—H23B	109.5
C8—C9—H9	119.4	O1—C23—H23C	109.5
C11—C10—C9	119.31 (16)	H23A—C23—H23C	109.5
C11—C10—H10	120.3	H23B—C23—H23C	109.5
			
C14—C1—C2—N2	−177.16 (16)	C9—C8—C13—C14	175.09 (14)
C15—C1—C2—N2	−3.0 (2)	C7—C8—C13—C14	−6.4 (2)
C14—C1—C2—C3	1.8 (2)	C2—C1—C14—C5	−1.9 (2)
C15—C1—C2—C3	175.94 (15)	C15—C1—C14—C5	−175.51 (15)
N2—C2—C3—C4	178.21 (17)	C2—C1—C14—C13	173.29 (15)
C1—C2—C3—C4	−0.7 (2)	C15—C1—C14—C13	−0.3 (3)
N2—C2—C3—C16	−0.9 (3)	C4—C5—C14—C1	1.0 (2)
C1—C2—C3—C16	−179.86 (15)	C6—C5—C14—C1	176.69 (15)
C2—C3—C4—C5	−0.1 (3)	C4—C5—C14—C13	−174.42 (16)
C16—C3—C4—C5	179.01 (16)	C6—C5—C14—C13	1.3 (2)
C2—C3—C4—C17	−177.76 (15)	C12—C13—C14—C1	28.1 (2)
C16—C3—C4—C17	1.4 (2)	C8—C13—C14—C1	−150.04 (16)
C3—C4—C5—C14	0.0 (3)	C12—C13—C14—C5	−156.66 (16)
C17—C4—C5—C14	177.51 (16)	C8—C13—C14—C5	25.2 (2)
C3—C4—C5—C6	−175.50 (16)	C5—C4—C17—C22	70.5 (2)
C17—C4—C5—C6	2.0 (3)	C3—C4—C17—C22	−111.93 (18)
C4—C5—C6—C7	132.25 (17)	C5—C4—C17—C18	−112.04 (19)
C14—C5—C6—C7	−43.4 (2)	C3—C4—C17—C18	65.5 (2)
C5—C6—C7—C8	58.90 (18)	C22—C17—C18—C19	2.4 (2)
C6—C7—C8—C9	142.75 (15)	C4—C17—C18—C19	−175.12 (15)
C6—C7—C8—C13	−35.7 (2)	C17—C18—C19—C20	0.5 (2)
C13—C8—C9—C10	−0.3 (2)	C23—O1—C20—C19	−160.73 (14)
C7—C8—C9—C10	−178.80 (15)	C23—O1—C20—C21	19.5 (2)
C8—C9—C10—C11	3.0 (2)	C18—C19—C20—O1	177.14 (14)
C9—C10—C11—C12	−2.2 (2)	C18—C19—C20—C21	−3.1 (2)
C10—C11—C12—C13	−1.4 (2)	O1—C20—C21—C22	−177.48 (15)
C11—C12—C13—C8	4.0 (2)	C19—C20—C21—C22	2.8 (2)
C11—C12—C13—C14	−174.11 (15)	C20—C21—C22—C17	0.2 (2)
C9—C8—C13—C12	−3.2 (2)	C18—C17—C22—C21	−2.7 (2)
C7—C8—C13—C12	175.35 (15)	C4—C17—C22—C21	174.76 (15)

### Hydrogen-bond geometry (Å, °)

**Table:**

*D*—H···*A*	*D*—H	H···*A*	*D*···*A*	*D*—H···*A*
N2—H1···O1^i^	0.89 (1)	2.21 (1)	3.0307 (19)	154.(2)
N2—H2···N1^ii^	0.88 (1)	2.33 (1)	3.115 (2)	149.(2)

Symmetry codes: (i) *x*−1, −*y*+1/2, *z*−1/2; (ii) −*x*−1, −*y*, −*z*.
